# A Comparison of Shear Bond Strengths of Metal and Ceramic Brackets using Conventional Acid Etching Technique and Er:YAG Laser Etching

**DOI:** 10.5681/joddd.2014.005

**Published:** 2014-03-05

**Authors:** Sogra Yassaei, Reza Fekrazad, Neda Shahraki, Mahdjoube Goldani Moghadam

**Affiliations:** ^1^Associate Professor, Department of Orthodontics, Faculty of Dentistry, Shahid Sadoughi University of Medical Sciences, Yazd, Iran; ^2^Department of Dentistry, - Laser Research Center, Tehran University of Medical Sciences, Tehran, Iran; ^3^Assistant professor, Department of Orthodontics, Faculty of Dentistry, Zahedan University of Medical Sciences, Zahedan, Iran; ^4^Postgraduate Student, Dental research center, Faculty of Dentistry, Birjand University of Medical Sciences, Birjand, Iran

**Keywords:** Er:YAG laser, etching, laser, orthodontic

## Abstract

***Background and aims.*** The aim of this study was to compare shear bond strength (SBS) of metal and ceramic brackets bonded to enamel using acid versus Er:YAG laser etching.

***Materials and methods.*** Eighty premolars were divided into 4 groups: AM (acid etching/ metal brackets), AC (acid etching/ ceramic brackets), LM (laser etching/ metal brackets) and LC (laser etching/ ceramic brackets). Enamel condition-ing was done using acid in AC and AM and Er:YAG laser in LC and LM. Brackets were debonded with a Dartec machine and the SBSs were determined. Adhesive remnant index was evaluated under a stereomicroscope. Two additional teeth were conditioned with acid and laser for scanning electron microscopy examination. Comparisons of SBS value were done by ANOVA test.

***Results.*** statistical analyses showed that SBSs of acid groups were significantly higher than that of laser groups, but dif-ferences between SBS values of AC/ AM and LC/LM were not significant. SEM examination revealed different etching pattern.

***Conclusion.*** Low power Er:YAG laser etching offers clinically acceptable SBS which besides its other superiorities to acid etching can be an appropriate alternative for bonding of ceramic brackets.

## Introduction

It is near two decades that orthodontic bracket bonding has been done by means of acid etching technique. Although this method has been extensively accepted by orthodontists,^1^ decalcification of enamel adjacent to brackets and consequent increased caries risk are known disadvantages of acid etch technique, more over it requires drying of enamel surface which is important in increasing the bond strength of brackets.^2^ In recent years there has been an increasing interest in lasers for medical and dental applications and various lasers have been designed to address different requirements.

Lasers have been used in orthodontics for conditioning of enamel and nonenamel tooth surfaces,^3-6^ composite removal from bracket base for recycling of brackets,^7-9^ debonding of ceramic brackets,^10-12^and curing.^13^ Laser irradiation causes thermal changes on the enamel surface and produces irregular porosities similar to acid etching,^14^ which are 10-20 µm in depth and can be used for conditioning of enamel and bonding procedures.^15^ Alteration in calcium to phosphorus ratio and decreased carbonate to phosphate percentage caused by laser irradiation to the enamel surface as well as reduction of water and organic content increase tooth resistance to caries.^16-18^ Er:YAG laser with wavelength of 2940 nm poses high absorption rate for water and hydroxyapatite; by careful control of radiation parameters the subsurface fissuring and subsequent adverse effect on adhesion (which are known disadvantages of lasers if irradiation parameters are not carefully controlled) are avoided.^19-20^

If adequate bond strength for bracket bonding can be provided by laser etching, it can be an alternative to acid etching technique. Previous studies offer contradictory findings about bond strength of brackets following laser etching in comparison with acid etching.^4,5,20-23^

The objective of this study was to compare efficiency of laser etching versus conventional acid etching in terms of resultant shear bond strength. According to the objectives of our study, the null hypothesis assumed that there were statistically significant differences between SBS values and mode of bond failure of stainless steel and ceramic brackets bonded to enamel using conventional acid etching technique or laser etching method.

Etching process provides mechanical retention for bonding of brackets. Metal brackets rely on mechanical retention which is conventionally provided by their mesh gauze,^24^ but ceramic brackets bond to enamel by two different mechanisms; mechanical retention and chemical bonding.^24^ Therefore, bond strength of ceramic brackets is also affected by chemical bonding. Since we hypothesized that different methods of etching might have different influences on SBS of stainless steel and ceramic brackets, we added the variable of bracket type and made the comparison between these two types. This comparison was made in attempt to decide about the best etching technique for each type of brackets. In addition, regarding the potential harmful thermal effect of lasers on dental pulp, we used laser etching at lower power compared to previous studies.^4,5,20-23^

## Materials and Methods

According to similar studies and by considering 5% for the first order error and 80% as the test power, it was determined that at least 20 teeth in each of the groups were required to achieve a minimum of 6 units difference in bond strength. Eighty human premolars which were extracted for orthodontic purposes, free of caries, fractures and enamel surface defects were collected and immersed in 0.5% sodium hypochlorite for infection control and stored in normal saline until start of the study. Before etching procedure the buccal surfaces of teeth were cleaned using non fluoridated pumice, then washed and dried.

The teeth were randomly divided into 4 groups of 20 teeth named AM (acid etching/ metal brackets), AC (acid etching/ ceramic brackets), LM (laser etching/ metal brackets), LC (laser etching/ ceramic brackets) according to conditioning method and type of brackets which were used. Metal brackets used in this study were stainless steel standard edgewise premolar brackets (Dentaurum Company, Ispringen, Germany). The ceramic brackets used were Fascination (Dentaurum, Ispringen, Germany), polycrystalline ceramic brackets which provide chemical retention. The ceramic brackets were also premolar brackets.

###  Acid Etching Procedure

The teeth in groups AM and AC were conditioned with a 37% phosphoric acid gel for 30 seconds. Following application of the acid, the buccal surfaces were rinsed completely for 20 seconds and dried with oil and moisture free air until the frosty white appearance was achieved.

###  Laser Etching Procedure 

The buccal surfaces were etched using laser irradiation in groups LM and LC. For this purpose an Er:YAG laser (KEY Laser 3+, KaVo Dental Corporation, Biberach , Germany) was used at 80 mJ, 15Hz for 10 seconds. These irradiation parameters of laser system were determined on the basis of pilot study. Average power output was 1.2 W and the laser operated at pulse mode. The 2060 handpiece (KaVo Dental Corporation, Biberach, Germany) of system was used at distance of 20 mm perpendicular to the buccal surfaces in swiping movement to etch the teeth. The buccal surfaces irradiated in defocused mode at an area slightly larger than bonding area. A water spray which laser system was equipped with was used for cooling of the teeth. After etching the teeth were dried with oil and moisture free air for 20 seconds.

###  Bracket Bonding

After etching process with acid (in AM and AC groups) or laser (in LM and LC groups), a thin adhesive resin layer (Resilience Ortho Technology, Tampa, Florida, USA) was applied on the teeth surfaces by means of a brush. The brackets were positioned centrally on the teeth surfaces and bonded using composite resin (Resilience Ortho Technology, Tampa, Florida, USA). Following removal of excessive composite resin with a dental explorer, adhesive was cured using Bluephase C8 light- emitting diode (LED) (Ivoclar Vivadent, Schaan, Liechtenstein) irradiating a light intensity of 650 Mw/ cm^2^ for 20 seconds (5 seconds for each of occlusal, gingival, mesial and distal direction).

After bracket bonding, the teeth were thermocycled in water between 5°C and 55°C for 500 cycles (30 seconds in 5°C water and 30 seconds in 55°C water) to simulate oral environment and make the study closer to the clinical situation.

### Bracket Debonding

After bonding procedure the teeth were mounted in blocks of self curing acrylic resin at the level of 1mm below the CEJ (cemento enamel junction) to stabilize specimens in a Dartec testing machine (Dartec, Zwick Roell Group, UK). The teeth were positioned in the acrylic blocks in a manner that bases of brackets were perpendicular to horizontal level.

In order to prevent from thermal changes following setting procedure of the acrylic resin the teeth were immersed in water for 10 minutes. The SBS was evaluated by the Dartec testing machine with a cross head speed of one mm per minute until bond failure, and the force needed to achieve bond failure was recorded in newtons. Shear bond strength was then calculated by dividing the values of force by the bracket base area which was 12 mm^2^ and was reported in MPa.

$$\text{Shear bond strength (Mpa)}=\frac{\text{Shear bond strenght (newtons)}}{{\text{Bracket base area (mm}}^{\text{2}}\text{)}}$$

### Residual Adhesive 

After debonding procedure all teeth were examined under a stereomicroscope at ×10 magnification to assess residual adhesive remaining on the enamel and the sites of bond failure between the enamel, resin and bracket base. The adhesive remnant index (ARI) which was introduced by Bishara and Trulove^25^ was used to evaluate the amount of adhesive left on the teeth surfaces:

Score 1: All the adhesive remained on the teeth

Score 2: More than 90% of the adhesive remained on the teeth

Score 3: Between 10-90% of the adhesive remained on the teeth

Score 4: Less than 10% of the adhesive remained on the teeth

Score 5: No adhesive remained on the teeth

SEM (scanning electron microscope) examination

Two additional teeth were etched; one with the 37% phosphoric acid and the other was lased according to the protocol described before. After special gold treatment of the enamel surfaces to make them ready for SEM examination, the teeth were evaluated under an electron microscope (VEGA, TESCAN Co. Czech Republic) to determine the pattern of etching.

###  Statistical Analysis

Descriptive statistics including mean and standard deviation (SD) of SBS values were calculated by means of statistical package for social sciences (SPSS) (SPSS for windows, release 10.0.0, Chicago, III). The Kolmogorov-Smirnov test showed normal distribution of data. The ANOVA and Tukey tests were used for multiple comparisons of SBS amounts between the groups. P < 0.05 was considered significant for all statistical tests.

## Results

###  Shear Bond Strength

The calculation of SBS was done by means of dividing the force needed to cause bond failure by bracket base area. Descriptive statistics including mean and standards deviation are presented in Table 1.

**Table 1:** Descriptive statistics including mean shear bond strength (MPa) and SD of study groups

Group	Number	Mean	SD	P value
AM	20	30.45	1.63	< 0.001
AC	20	32.25	1.37	
LC	20	20.12	3.43	
LM	20	18.32	3.00	
AM (acid etching/ metal brackets), AC (acid etching/ ceramic brackets), LM (laser etching/ metal brackets) and LC (laser etching/ ceramic brackets).				

According to the ANOVA test (Table 1) there were significant differences between mean SBS values of study groups (P < 0.05). Multiple comparisons were done between groups by means of the Tukey post hoc test (Table 2) which showed significantly higher SBS values for acid etched groups (AC and AM) compared to lased groups (LC and LM). The difference in the SBS of the teeth conditioned with acid and bonded with either of metal or ceramic brackets (AC versus AM) was not statistically significant (P > 0.05), similarly comparison between SBS values of LC and LM groups revealed no significant difference (P > 0.05).

**Table 2:** Multiple comparisons of shear bond strength (SBS) values between groups using Tukey test

Group	AM		AC		LM		LC	
	P- value	Sig	P- value	Sig	P- value	Sig	P- value	Sig
AM	-	-	0.118	NS	0.000	*	0.000	*
AC	-	-	-	-	0.000	*	0.000	*
LM	-	-	-	-	-	-	0.117	NS
LC	-	-	-	-	-	-	-	-
AM (acid etching/ metal brackets), AC (acid etching/ ceramic brackets), LM (laser etching/ metal brackets) and LC (laser etching/ ceramic brackets). NS: not significant; ^*^: P- value < 0.05								

Maximum amount of SBS was belonged to AC group (32.25 MPa), followed in decreasing order by AM (30.45 MPa), LC (20.12 MPa) and LM (18.32 MPa).

###  ARI Scores

Frequencies of ARI scores for each of the groups along are shown in Table 3. The results showed higher frequency of ARI scores of 3 in AM, 5 in AC, 2 in LM and 3 in LC.

**Table 3:** Distribution of adhesive remnant score (1 5)on enamel surface in study groups

Group	1		2		3		4		5	
	n	%	n	%	n	%	n	%	n	%
AM	0	0	1	5	13	65	5	25	1	5
AC	0	0	0	0	3	15	7	35	10	50
LM	7	35	8	40	3	15	2	10	0	0
LC	1	5	3	15	10	50	6	30	0	0
AM (acid etching/ metal brackets), AC (acid etching/ ceramic brackets), LM (laser etching/ metal brackets) and LC (laser etching/ ceramic brackets).										

### SEM Examination

**Figure 1.**
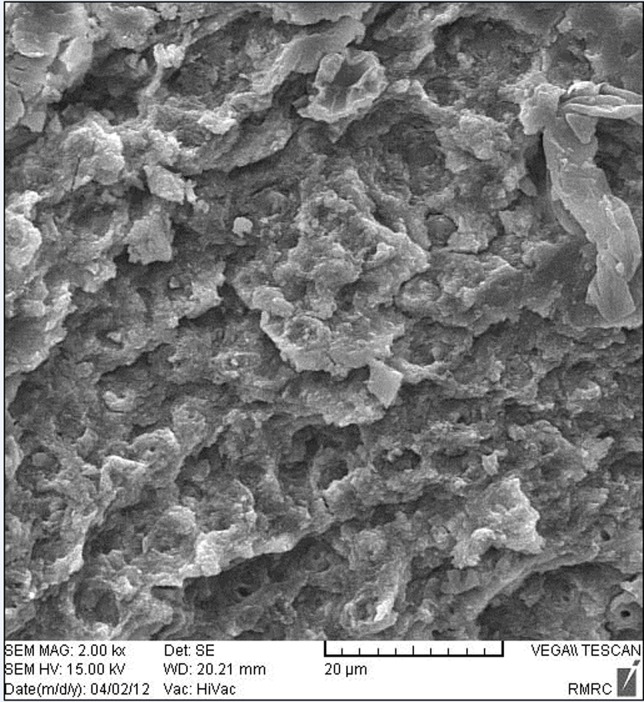
Enamel surface after 80 mJ of Er:YAG laser irradiation for 10 seconds (×2000).

**Figure 2.**
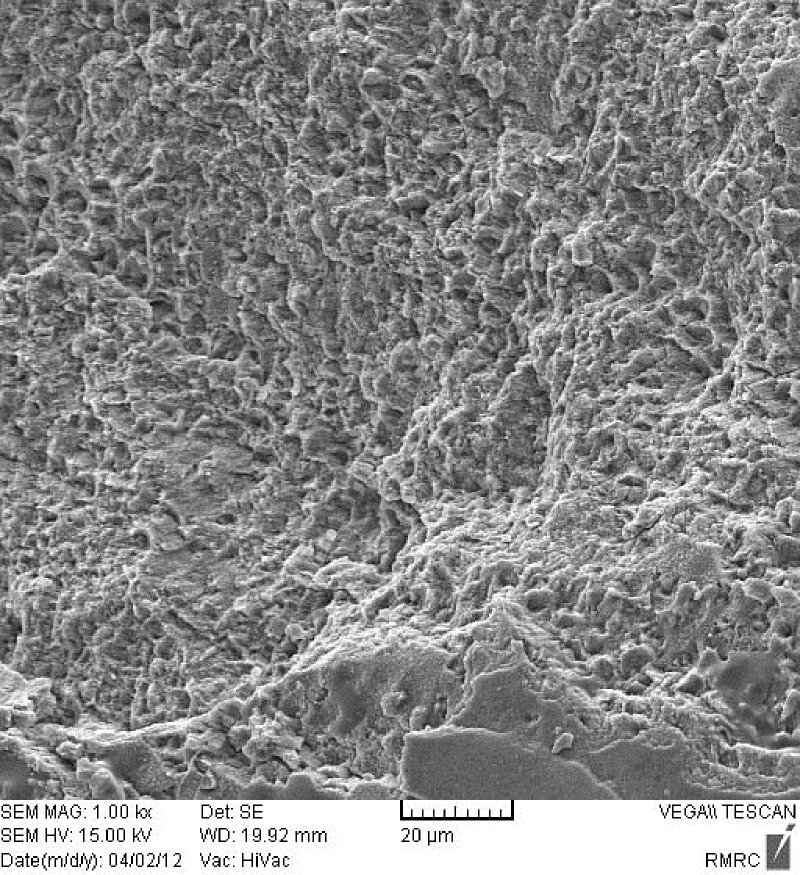
Enamel surface after conventional acid etching (×1000).

**Figure 3.**
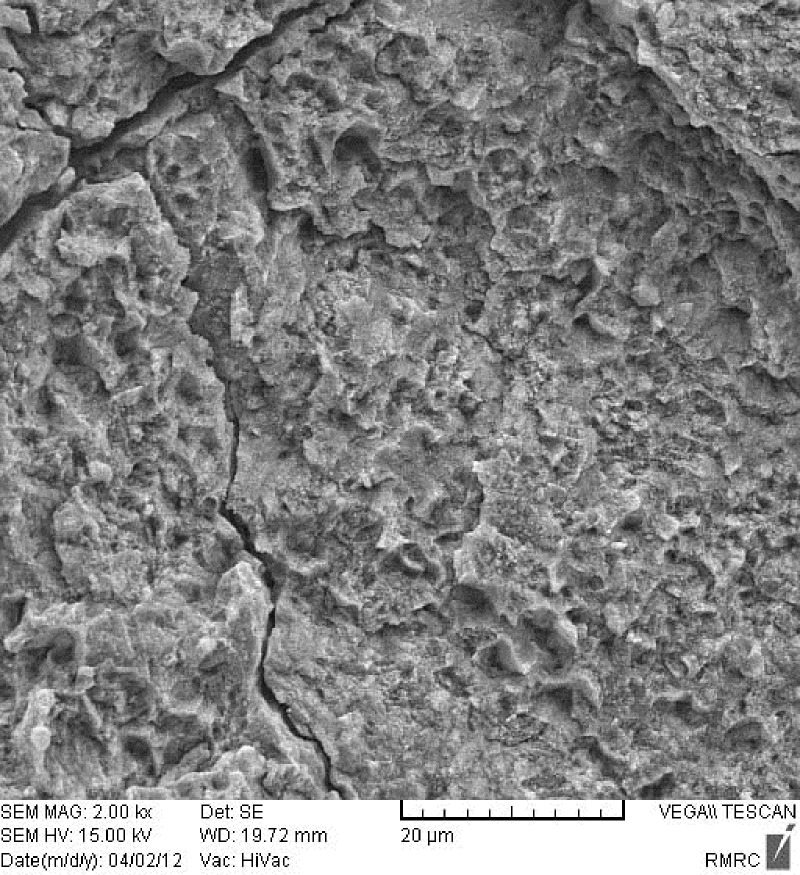
Enamel surface after conventional acid etching. Note the cracks on enamel surface (×1000).

Figure 1 shows an enamel surface after the Er:YAG laser irradiation which is in accordance with type 3 etching pattern described by Silverstone et al ^14^. They have shown that in vitro exposure of enamel to acid solutions produced three basic etching patterns. In type 1 etching pattern, prism core material was preferentially dissolved while the prism peripheries remained relatively unaffected. In type 2 etching pattern the peripheral regions of prisms were removed leaving prism cores relatively intact. Etching pattern of type 3 was characterized by a more random pattern with areas corresponded to types 1 and 2 together. They also observed regions in which the pattern of etching could not be related to prism morphology. As shown in Figure 2, acid etched pattern of conventional acid etching technique produced enamel surface resembled type 1 pattern of etching according to Silverstone et al.^14^ No fracture or crack is observed in the lased enamel surface, while evaluation of the acid etched enamel shows cracks in some regions (Figure 3).

## Discussion

Extensive researches have been carried out on the issue of bracket bonding to enamel. Despite different alternatives to acid etching, this method is probably the best method of bonding resins to enamel.^1,19^ Demineralization of enamel caused by acid application can make it susceptible to long-term acid attack and carries, especially when air bubbles and saliva contamination disturb the resin penetrance and plaque accumulation adjacent to the brackets aggravates the condition.^26-28^

Alternatives to phosphoric acid etching such as application of Maleic and Polyacrylic acids as well as sandblasting have been suggested, but weak bond strength resulted from these methods is a limitation of using them.^27,28^ Another alternative to phosphoric acid etching which has been recently introduced to dentistry is laser etching. Contrary to the acid etching, the lased surface is fissured and less homogenous. Enamel prisms orientation and anisotropic nature of enamel can be possible explanation for fissuring.^19,20^ Alteration of calcium to phosphorus ratio, reduction of carbonate, water and organic content and pyrophosphate formation following laser etching decrease the caries risk,^16-18^and this laser induced caries resistance is of great importance in orthodontics.^16,19^

The results of our study showed that SBS values of brackets bonded with laser etching were significantly lower than that of acid etching. Reynolds^29^suggested that bond strengths ranged from 6 to 8 MPa provided clinically acceptable bonding. Therefore, obtained SBS values for LM and LC groups in our study are still clinically acceptable strengths for orthodontic bracket bonding.

It was also shown that mean SBSs obtained in acid etching groups were higher than laser etching groups which is in accordance with the results of studies done by von Fraunhofer et al,^15^ Corpas-Pastor et al,^30^ and Martinez-Insua et al,^31^ but is different from Findings of Visuri et al^32^ and Keller & Hibst.^19^

Lower bond strengths of lased groups (LM and LC) in our study can be attributed, at least in part, to the pattern of laser etching which was affected by hand control of sweeping motion of laser beam and it can be a possible explanation for weak etching of enamel by laser. There are, however, other possible explanations; enamel micro pores, cracks, craters and melted bubbles produced during laser etching procedure,^33^ as well as laser power and superficial energy exerted by laser are other interfering factors.^34^ Although no fracture or crack was observed in lased enamel surface in the SEM examination of the present study, it should be interpreted with caution because the SEM evaluation in this study was made on the basis of qualitative description. Furthermore, single specimen from each of laser etching and acid etching groups was evaluated which was not representative of the whole group. Lower bond strength obtained by the laser etching can mask its advantages such as reducing caries risk.

The higher ranges of SDs seen in the laser groups compared to the acid groups are probably the result of hand control of swiping movement of the laser beam which might cause weekly standardized etching pattern. Differences in intrinsic nature of the teeth gathered from different people as well as different time of storage and environmental effects could influence SDs in all study groups. To control these problems, use of animal teeth can be helpful because numerous teeth can be collected from an animal.

From clinical point of view laser aided enamel conditioning makes the teeth surfaces resistant to acid attack and needs less isolated field to obtain adequate bond strength. Regarding these advantages we can consider it a useful technique wherever achieving higher bond strength is not critical, which that is the case when we are using ceramic brackets. Debonding of ceramic brackets is difficult and imposes the risk of enamel fracture and crazing.^24^ This is especially important when chemical bonding of ceramic brackets with silane coupling agent is provided. Although the SBSs of metal and ceramic brackets bonded to conventional acid etched enamel were comparable in our study, chemically bonded ceramic brackets have been said to produce excessive bond strength.^35^ When clinicians select this type of bracket, reducing the bond strength is preferred. Because of mentioned advantages of laser etching, it can be the method of choice when reducing bond strength is favorable.

We used laser etching at lower power compared to the previous studies. Thermal effects of lasers can cause injury to the pulp tissue. Zach and Cohen^36^ in their study to evaluate the effect of externally applied heat on the pulp tissue concluded that no pulpal injury occurred when the maximum intrapulpal temperature rise stayed below 5.5 degrees C. Liu et al^37^ evaluated histomorphological effects of Nd:YAG on the pulp tissue during laser debonding of ceramic brackets. They found that pulp tissue of the teeth exposed to laser irradiation for 5 minutes showed mild capillary dilation and concluded that the Nd:YAG laser of high energy could cause pulp tissue injury during debonding while using the Nd:YAG laser of lower energy could debond brackets effectively without imposing the risk of irreversible injury to the pulp.

Residual adhesive assessment was done according to the adhesive remnant index (ARI) introduced by Bishara and Trulove.^25^ The amounts of adhesive remnant can be evaluated with both qualitative and quantitative methods. For qualitative evaluation 4-point scale of Artun and Bergland^38^ (original method) and 5-point scale of Bishara and Trulove^25^ (modified method) have been used extensively in the literature. We assumed that the modified ARI provided a more precise definition of site of bond failure since it was scored in smaller range; therefore the amount of residual adhesive was assessed according to 5-point scale version in this study.

The results of ARI scoring showed that bond failure in AM and LC groups occurred mostly in a manner that between 10 to 90% of adhesive remained on the enamel surface, while bond failure in AC group often (50%) left no adhesive remnant on the enamel surface. In LM group most of the time (40%) more than 90% of the adhesive remained on the teeth. However, in the light of wide range of score 3 of ARI interpreting the results cannot be done accurately. Although the issue of preferable site of bond failure has been controversial up to now, some authors demonstrated that bond failure at the bracket- adhesive surface was better, since it reduced the risk of enamel fracture and crazing during debonding procedure, especially for ceramic brackets.^39^ Others believed that bond failure at enamel- adhesive interface was preferred since it left lesser residual adhesive remnants and consequently fewer chair time was needed to remove them.^25^

SEM Examination of the etched enamel specimens showed etching pattern which resembled type 3 etching pattern in the lased enamel surface and type 1 in acid etched enamel. Basaran et al^5^ who examined their specimens under electron microscope found type 3 etching pattern according to Silverstone^14^ for orthophosphoric acid etching and a similar pattern by a 2-W Er,Cr:YSGG irradiation, while the etching pattern of 1-W laser irradiation was in accordance with type 1 etching pattern. Usumez et al^22^ who evaluated enamel surface characteristics of the acid etched and Er,Cr:YSGG lased enamel found type 2 and type 3 etching pattern respectively, however it seems that there is no well defined relationship between etching pattern of enamel and the resultant shear bond strength.^40^ Observation of cracks in the etched enamel which is not a routine feature is likely due to processing of the samples for SEM.

## Conclusion

From the present study it can be concluded that:

1. Mean shear bond strength of brackets bonded to enamel using phosphoric acid etching is significantly higher than brackets bonded by means of Er: YAG laser (80 mJ, 15 Hz, 10 seconds) etching.
2. The modes of bond failure of stainless steel and ceramic brackets bonded to enamel using conventional acid etching technique or laser etching method were different.
3. SEM examination showed etching pattern resembling type 3 and type1 (according to Silverstone et al. classification) for laser and acid etching respectively.
